# Air temperature and mean radiant temperature data, collected and simulated across a radiantly-heated high-bay laboratory

**DOI:** 10.1016/j.dib.2020.105192

**Published:** 2020-02-03

**Authors:** Hongshan Guo, Maria Ferrara, James Coleman, Mauricio Loyola, Forrest Meggers

**Affiliations:** a Andlinger Center for Energy and the Environment, Princeton University, Princeton, NJ, 08544, United States; b School of Architecture, Princeton University, Princeton, NJ, 08544, United States; c Politecnico di Torino, Torino, Italy

**Keywords:** Mean radiant temperature, Thermal comfort, Building simulation, Radiant temperature, Human-centric control

## Abstract

To better understand the extent of how the air temperature and mean radiant temperature may vary both spatially and temporally in a radiantly heated space, we conducted a seven-day experiment in the architectural laboratory at School of Architecture, Princeton University. The primary intent of this paper was to decouple the measurement of the air temperature and mean radiant temperature. We collected a large dataset that shows temporal and spatial variations. To do so, we used non-contact infrared thermometer to measure the surface temperatures of the surrounding surfaces inside the laboratory. The geometry of the laboratory is simplified into a box, the corresponding view factor from every point within the box can be calculated towards each internal surface. These view factors are then combined with the measured surface temperatures to produce mean radiant temperatures. This spatial mean radiant temperature distribution was then compared with the air temperature distribution measured by the air temperature sensors suspended from the ceiling of the laboratory. We believe making these data available will help future researchers working on similar problems to develop protocols than the state-of-the-art measurement techniques observed among different thermal comfort or radiant heat transfer research.

**Table:** Specifications Table

Subject	Energy Engineering and Power Technology
Specific subject area	We measured the spatial air temperature, relative humidity and deducted the corresponding mean radiant temperatures from surface temperatures collected through non-contact infrared thermometers. This attempt builds on the existing state-of-the-art measurement techniques of the indoor environment where the air temperatures and the mean radiant temperatures are collected independently instead of having to correct the convection readings out from integral readings (such as that from globe thermometers, where air temperature and mean radiant temperatures are simultaneously measured).
Type of data	Image Figure Video Comma-Separated Values (CSV) files
How data were acquired	Air temperature and relative humidity collected with DHT22s; Surface temperatures were collected through non-contact infrared thermometer Melexis® MLX90614; Water inlet/outlet temperature at different system components collected through single-ended NTC 1% thermistors.
Data format	Raw Filtered
Parameters for data collection	The data were collected continuously throughout the period of the experimental measurements.
Description of data collection	Data were collected through small Wi-Fi enabled developer boards that captures the data through the sensor and publish them to the REST API server. In the meantime. We used Python scripts to scrape the REST API URL to download the freshly published data down to a local InfluxDB server, which we monitored through a Grafana visualization interface.
Data source location	City/Town/Region: Princeton, New Jersey Country: United States Latitude and longitude (and GPS coordinates) for collected samples/data: 40°20′37.1″N 74°38′59.5″W
Data accessibility	Uploaded with the article
Related research article	Authors: Hongshan Guo, Maria Ferrara, James Coleman, Mauricio Loyola, Forrest Meggers Title: Simulation and measurement of air temperatures and mean radiant temperatures in a radiantly heated indoor space Journal: Energy https://doi.org/10.1016/j.energy.2019.116369

**Table:** 

**Value of the Data** • The published data presents pioneering effort of joined investigation of spatial air temperature and mean radiant temperature measurement of a radiantly heated open space; • Mechanical and HVAC engineers as well as architects can benefit from the understanding of how the air temperature and mean radiant temperature varies across space and time, which could guide future system design; • The spatial and temporal variation of both the air temperature and mean radiant temperature can be used by future studies to understand the level of granularity to questions and doubts in designing not only radiant systems, and systems with larger radiant surfaces – full glass façade, etc. • The data could also provide valuable insights on future evaluation of mean radiant temperature as it highlights the importance of separating the measurement of air temperature from the mean radiant temperature.

## 1 Data

We are including two sets of data with this submission: the first set contains the raw data files downloaded directly from the InfluxDB server – which includes the air temperature, surface temperature and corresponding system components readings (e.g. temperature at the surface of the radiant floor). The second set is the filtered data where the time stamps are consistent throughout the time of the measurement, mean radiant temperature approximated from the view factors calculated. Both datasets are in csv formats, while the latter – plotted against each other – is easier to interpret and compare between one another. In addition, we have included a video of the mean radiant temperature changing over time.

## 2 Experimental design, materials, and methods

As was explained above, the data-scraping of the REST API supplied by the developer board maker, Particle, is achieved with Python script that monitors the account ssh events. With each new event published at the RESTP API, the script recognizes the json and parse it into identifiers such as time, device name, variable names and variable values. The values are then written through Python into the InfluxDB as entries with legible time stamps. On the front end, the Grafana interface talks only with InfluxDB and can be used with InfluxDB's own query language to either show the data within a specific time range – or a specific device/variable. It was also possible to export datasets directly from Grafana, where the time stamps will be consistent since it re-creates the time stamps using group-by command of different sizes of time interval (e.g. 1 m, 5 m, 15 m, etc.).

**Fig. 1:**
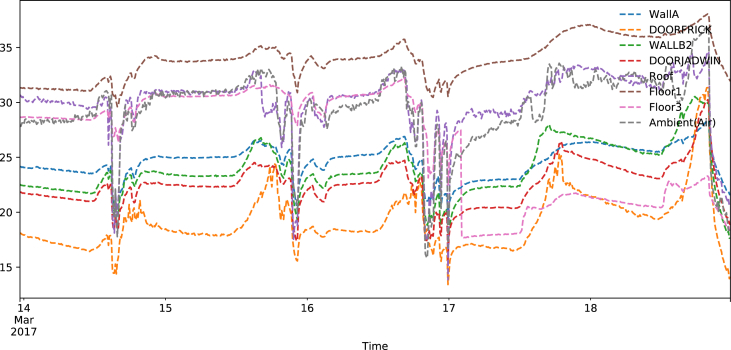
Surface temperature data from the first data set plotted against period of experiment.

**Fig. 2:**
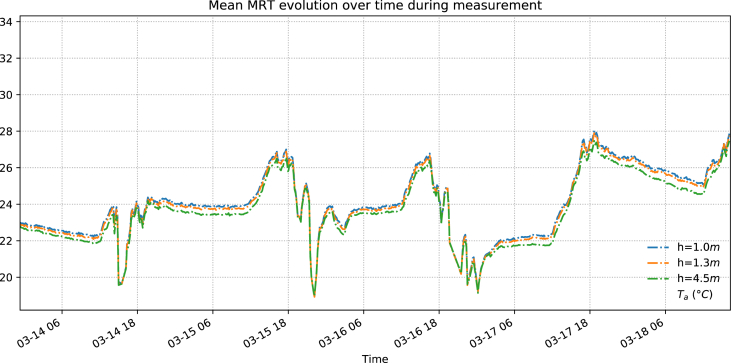
From the second dataset: Close-up of calculated MRT plotted against air temperature.

Using these smoothed data, we created Fig. 1 to demonstrate the variations of surface temperatures during the experiment. These raw readings from the Melexis® readers are plotted against the air temperature collected from the DHT22s (plotted as grey dotted line) in Fig. 1. We calculated the corresponding view factors of the respective surfaces using methods outlined in our previous work [1], stepping beyond what Fanger in 1970 [2] as well as some more recent literature [3] and obtained Fig. 2, where we plot the respective temporal change of mean radiant temperature at different locations.

## References

[1] Guo Hongshan, Ferrara Maria, Coleman James, Loyola Mauricio, Meggers Forrest (2019). Simulation and measurement of air temperatures and mean radiant temperatures in a radiantly heated indoor space. Energy.

[2] Fanger P.O. (1970). Thermal Comfort: Analysis and Applications in Environmental Engineering.

[3] Wang Yan, Meng Xi, Zhang Lili, Liu Yulan, Long Enshen (2014). Angle factor calculation for the thermal radiation environment of the human body, proceedings of the 8^th^ International symposium on heating, ventilation and air conditioning.

